# Predicting disease risks from highly imbalanced data using random forest

**DOI:** 10.1186/1472-6947-11-51

**Published:** 2011-07-29

**Authors:** Mohammed Khalilia, Sounak Chakraborty, Mihail Popescu

**Affiliations:** 1 Department of Computer Science, University of Missouri, Columbia, Missouri, USA; 2 Department of Statistics, University of Missouri, Columbia, Missouri, USA; 3 Department of Health Management and Informatics, University of Missouri, Columbia, Missouri, USA

## Abstract

**Background:**

We present a method utilizing Healthcare Cost and Utilization Project (HCUP) dataset for predicting disease risk of individuals based on their medical diagnosis history. The presented methodology may be incorporated in a variety of applications such as risk management, tailored health communication and decision support systems in healthcare.

**Methods:**

We employed the National Inpatient Sample (NIS) data, which is publicly available through Healthcare Cost and Utilization Project (HCUP), to train random forest classifiers for disease prediction. Since the HCUP data is highly imbalanced, we employed an ensemble learning approach based on repeated random sub-sampling. This technique divides the training data into multiple sub-samples, while ensuring that each sub-sample is fully balanced. We compared the performance of support vector machine (SVM), bagging, boosting and RF to predict the risk of eight chronic diseases.

**Results:**

We predicted eight disease categories. Overall, the RF ensemble learning method outperformed SVM, bagging and boosting in terms of the area under the receiver operating characteristic (ROC) curve (AUC). In addition, RF has the advantage of computing the importance of each variable in the classification process.

**Conclusions:**

In combining repeated random sub-sampling with RF, we were able to overcome the class imbalance problem and achieve promising results. Using the national HCUP data set, we predicted eight disease categories with an average AUC of 88.79%.

## Background

The reporting requirements of various US governmental agencies such as Center for Disease Control (CDC), Agency for Health Care Quality (AHRQ) and US Department of Health and Human Services Center for Medicare Services (CMS) have created huge public datasets that, we believe, are not utilized to their full potential. For example, CDC http://www.cdc.gov makes available National Health and Nutrition Examination Survey (NHANES) data. Using NHANES data, Yu et al. [1] predicts diabetes risk using an SVM classifier. CMS http://www.cms.gov uses the Medicare and Medicaid claims to create the minimum dataset (MDS). Herbert et al. [2] uses MDS data to identify people with diabetes. In this paper we use the National Inpatient Sample (NIS) data created by AHRQ http://www.ahrq.gov Healthcare Utilization Project (HCUP), to predict the risk for eight chronic diseases.

Disease prediction can be applied to different domains such as risk management, tailored health communication and decision support systems. Risk management plays an important role in health insurance companies, mainly in the underwriting process [3]. Health insurers use a process called underwriting in order to classify the applicant as standard or substandard, based on which they compute the policy rate and the premiums individuals have to pay. Currently, in order to classify the applicants, insurers require every applicant to complete a questionnaire, report current medical status and sometimes medical records, or clinical laboratory results, such as blood test, etc. By incorporating machine learning techniques, insurers can make evidence based decisions and can optimize, validate and refine the rules that govern their business. For instance, Yi et al. [4], applied association rules and SVM on an insurance company database to classify the applicants as standard, substandard or declined.

Another domain where disease prediction can be applied is tailored health communication. For example, one can target tailored educational materials and news to a subgroup, within the general population, that has specific disease risks. Cohen et al. [5], discussed how tailored health communication can motivate cancer prevention and early detection. Disease risk prediction along with tailored health communication can lead to an effective channel for delivering disease specific information for people who will be likely to need it.

In addition to population level clinical knowledge, deidentified public datasets represent an important resource for the clinical data mining researchers. While full featured clinical records are hard to access due to privacy issues, deidentified large national public dataset are readily available [6]. Although these public datasets don't have all the variables of the original medical records, they still maintain some of their main characteristics such as data imbalance and the use of controlled terminologies (ICD-9 codes).

Several machine learning techniques were applied to healthcare data sets for the prediction of future health care utilization such as predicting individual expenditures and disease risks for patients. Moturu et al. [7], predicted future high-cost patients based on data from Arizona Medicaid program. They created 20 non-random data samples, each sample with 1,000 data points to overcome the problem of imbalanced data. A combination of undersampling and oversampling was employed to a balanced sample. They used a variety of classification methods such as SVM, Logistic Regression, Logistic Model Trees, AdaBoost and LogitBoost. Davis et al. [8], used clustering and collaborative filtering to predict individual disease risks based on medical history. The prediction was performed multiple times for each patient, each time employing different sets of variables. In the end, the clusterings were combined to form an ensemble. The final output was a ranked list of possible diseases for a given patient. Mantzaris et al. [9], predicted Osteoporosis using Artificial Neural Network (ANN). They used two different ANN techniques: Multi-Layer Perceptron (MLP) and Probabilistic Neural Network (PNN). Hebert et al. [2], identified persons with diabetes using Medicare claims data. They ran into a problem where the diabetes claims occur too infrequently to be sensitive indicators for persons with diabetes. In order to increase the sensitivity, physician claims where included. Yu et al. [1], illustrates a method using SVM for detecting persons with diabetes and pre-diabetes.

Zhang et al. [10], conducted a comparative study of ensemble learning approaches. They compared AdaBoost, LogitBoost and RF to logistic regression and SVM in the classification of breast cancer metastasis. They concluded that ensemble learners have higher accuracy compared to the non-ensemble learners.

Together with methods for predicting disease risks, in this paper we discuss a method for dealing with highly imbalanced data. We mentioned two examples [2,7] where the authors encountered class imbalanced problems. Class imbalance occurs if one class contains significantly more samples than the other class. Since the classification process assumes that the data is drawn from the same distribution as the training data, presenting imbalanced data to the classifier will produce undesirable results. The data set we use in this paper is highly imbalanced. For example, only 3.59% of the patients have heart disease, thus it is possible to train a classifier with this data and achieve an accuracy of 96.41% while having 0% sensitivity.

## Methods

### Dataset

The Nationwide Inpatient Sample (NIS) is a database of hospital inpatient admissions that dates back to 1988 and is used to identify, track, and analyze national trends in health care utilization, access, charges, quality, and outcomes. The NIS database is developed by the Healthcare Cost and Utilization Project (HCUP) and sponsored by the Agency for Healthcare Research and Quality (AHRQ) [6]. This database is publicly available and does not contain any patient identifiers. The NIS data contains discharge level information on all inpatients from a 20% stratified sample of hospitals across the United States, representing approximately 90% of all hospitals in the country [6]. The five strata for hospitals are based on the American Hospital Association classification. HCUP data from the year 2005 will be used in this paper.

The data set contains about 8 million records of hospital stays, with 126 clinical and nonclinical data elements for each visit (table 1). Nonclinical elements include patient demographics, hospital identification, admission date, zip code, calendar year, total charges and length of stay. Clinical elements include procedures, procedure categories, diagnosis codes and diagnosis categories. Every record contains a vector of 15 diagnosis codes. The diagnosis codes are represented using the *International Classification of Diseases, Ninth Revision, Clinical Modification *(ICD-9-CM). The International Statistical Classification of Disease is designed and published by the World Health Organization (WHO). The ICD-9 codes are alphanumeric codes, 3-5 characters long and used by hospitals, insurance companies and other facilities to describe health conditions of the patient. Every code represents a disease, condition, symptom, or cause of death. There are numerous codes, over 14,000 ICD-9 codes and 3,900 procedures codes.

**Table 1:** HCUP data elements

	Element Name	Element Description
**1**	AGE	Age in years at admission
**2**	AGEDAY	Age in days (when age > 1 year)
**3**	AMONTH	Admission month
**4**	ASOURCE	Admission source (uniform)
**5**	ASOURCEUB92	Admission source (UB-92 standard coding)
**6**	ASOURCE_X	Admission source (as received from source)
**7**	ATYPE	Admission type
**8**	AWEEKEND	Admission day is a weekend
**9**	DIED	Died during hospitalization
**10**	DISCWT	Weight to discharges in AHA universe
**11**	DISPUB92	Disposition of patient (UB-92 standard coding)
**12**	DISPUNIFORM	Disposition of patient (uniform)
**13**	DQTR	Discharge quarter
**14**	DRG	DRG in effect on discharge date
**15**	DRG18	DRG, version 18
**16**	DRGVER	DRG grouper version used on discharge date
**17**	DSHOSPID	Data source hospital identifier
**18**	DX1	Principal diagnosis
**19**	DX2	Diagnosis 2
**20**	DX3	Diagnosis 3
**21**	DX4	Diagnosis 4
**22**	DX5	Diagnosis 5
**23**	DX6	Diagnosis 6
**24**	DX7	Diagnosis 7
**25**	DX8	Diagnosis 8
**26**	DX9	Diagnosis 9
**27**	DX10	Diagnosis 10
**28**	DX11	Diagnosis 11
**29**	DX12	Diagnosis 12
**30**	DX13	Diagnosis 13
**31**	DX14	Diagnosis 14
**32**	DX15	Diagnosis 15
*33	DXCCS1	CCS: principal diagnosis
*34	DXCCS2	CCS: diagnosis 2
*35	DXCCS3	CCS: diagnosis 3
*36	DXCCS4	CCS: diagnosis 4
*37	DXCCS5	CCS: diagnosis 5
*38	DXCCS6	CCS: diagnosis 6
*39	DXCCS7	CCS: diagnosis 7
*40	DXCCS8	CCS: diagnosis 8
*41	DXCCS9	CCS: diagnosis 9
*42	DXCCS10	CCS: diagnosis 10
*43	DXCCS11	CCS: diagnosis 11
*44	DXCCS12	CCS: diagnosis 12
*45	DXCCS13	CCS: diagnosis 13
*46	DXCCS14	CCS: diagnosis 14
*47	DXCCS15	CCS: diagnosis 15
**48**	ECODE1	E code 1
**49**	ECODE2	E code 2
**50**	ECODE3	E code 3
**51**	ECODE4	E code 4
**52**	ELECTIVE	Elective versus non-elective admission
**53**	E_CCS1	CCS: E Code 1
**54**	E_CCS2	CCS: E Code 2
**55**	E_CCS3	CCS: E Code 3
**56**	E_CCS4	CCS: E Code 4
**57**	FEMALE	Indicator of sex
**58**	HOSPID	HCUP hospital identification number
**59**	HOSPST	Hospital state postal code
**60**	KEY	HCUP record identifier
**61**	LOS	Length of stay (cleaned)
**62**	LOS_X	Length of stay (as received from source)
**63**	MDC	MDC in effect on discharge date
**64**	MDC18	MDC, version 18
**65**	MDNUM1_R	Physician 1 number (re-identified)
**66**	MDNUM2_R	Physician 2 number (re-identified)
**67**	NDX	Number of diagnoses on this record
**68**	NECODE	Number of E codes on this record
**69**	NEOMAT	Neonatal and/or maternal DX and/or PR
**70**	NIS_STRATUM	Stratum used to sample hospital
**71**	NPR	Number of procedures on this record
**72**	PAY1	Primary expected payer (uniform)
**73**	PAY1_X	Primary expected payer (as received from source)
**74**	PAY2	Secondary expected payer (uniform)
**75**	PAY2_X	Secondary expected payer (as received from source)
**76**	PL_UR_CAT4	Patient Location: Urban-Rural 4 Categories
**77**	PR1	Principal procedure
**78**	PR2	Procedure 2
**79**	PR3	Procedure 3
**80**	PR4	Procedure 4
**81**	PR5	Procedure 5
**82**	PR6	Procedure 6
**83**	PR7	Procedure 7
**84**	PR8	Procedure 8
**85**	PR9	Procedure 9
**86**	PR10	Procedure 10
**87**	PR11	Procedure 11
**88**	PR12	Procedure 12
**89**	PR13	Procedure 13
**90**	PR14	Procedure 14
**91**	PR15	Procedure 15
**92**	PRCCS1	CCS: principal procedure
**93**	PRCCS2	CCS: procedure 2
**94**	PRCCS3	CCS: procedure 3
**95**	PRCCS4	CCS: procedure 4
**96**	PRCCS5	CCS: procedure 5
**97**	PRCCS6	CCS: procedure 6
**98**	PRCCS7	CCS: procedure 7
**99**	PRCCS8	CCS: procedure 8
**100**	PRCCS9	CCS: procedure 9
**101**	PRCCS10	CCS: procedure 10
**102**	PRCCS11	CCS: procedure 11
**103**	PRCCS12	CCS: procedure 12
**104**	PRCCS13	CCS: procedure 13
**105**	PRCCS14	CCS: procedure 14
**106**	PRCCS15	CCS: procedure 15
**107**	PRDAY1	Number of days from admission to PR1
**108**	PRDAY2	Number of days from admission to PR2
**109**	PRDAY3	Number of days from admission to PR3
**110**	PRDAY4	Number of days from admission to PR4
**111**	PRDAY5	Number of days from admission to PR5
**112**	PRDAY6	Number of days from admission to PR6
**113**	PRDAY7	Number of days from admission to PR7
**114**	PRDAY8	Number of days from admission to PR8
**115**	PRDAY9	Number of days from admission to PR9
**116**	PRDAY10	Number of days from admission to PR10
**117**	PRDAY11	Number of days from admission to PR11
**118**	PRDAY12	Number of days from admission to PR12
**119**	PRDAY13	Number of days from admission to PR13
**120**	PRDAY14	Number of days from admission to PR14
**121**	PRDAY15	Number of days from admission to PR15
**122**	RACE	Race (uniform)
**123**	TOTCHG	Total charges (cleaned)
**124**	TOTCHG_X	Total charges (as received from source)
**125**	YEAR	Calendar year
**126**	ZIPInc_Qrtl	Median household income quartile for patient's ZIP Code

Complete list of 126 HCUP data elements. The elements marked with "*" (rows 33-47) are the ones used in the classification as input variables.

*HCUP data elements used in the classification

**Figure 1:**
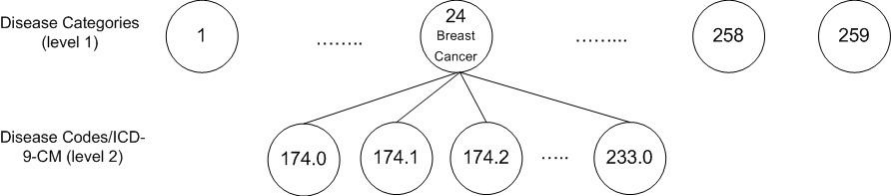
**Disease codes and categories hierarchical relationship**. This is a snap shot of the hierarchical relationship between the diseases and disease categories. For instance, disease category 49 (diabetes) has a children that are represented in disease codes (ICD-9-CM).

In addition, every record contains a vector of 15 diagnosis category codes. The diagnosis categories are computed using the Clinical Classification Software (CCS) developed by HCUP in order to categorize the ICD-9 diagnosis and procedure codes. CCS collapsed these codes into a smaller number of clinically meaningful categories, called diagnosis categories. Every ICD-9 code has a corresponding diagnosis category and every category contains a set of ICD-9 codes. We denote each of the 259 disease categories by a value between 1 and 259. In Figure 1 we show an example of disease category ("Breast cancer") and some of the ICD-9 codes included in it (174.0 -"Malignant neoplasm of female breast", 174.1-Malignant neoplasm of central portion of female breast", 174.2-Malignant neoplasm of upper-inner quadrant of female breast", 233.0-"Carcinoma in situ of breast and genitourinary system").

**Figure 2:**
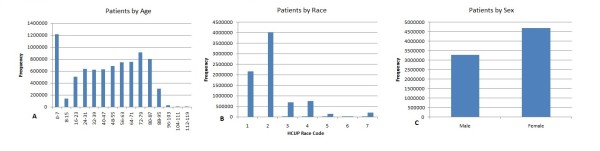
**Demographics of patients by age, race and sex for the HCUP data set**.

Demographics such as age, race and sex are also included in the data set. Figure 2 shows the distribution of patients across age, race and sex.

The data set is highly imbalanced. The imbalance rate ranges across diseases from 0.01-29.1%. 222 diagnosis categories occurs in less than 5% of the patients and only 13 categories occur in more than 10% of the patients. In table 2 we show the top 10 most prevalent disease categories and in table 3 we show some of the rarest diseases present in the 2005 HCUP data set.

**Table 2:** The 10 most prevalent diseases categories

Disease Category	Prevalence
Hypertension	29.1%
Coronary Atherosclerosis	27.65%
Hyperlipidemia	14.46%
Dysrhythmia	14.35%
Other Circulatory Diseases	12.02%
Diabetes mellitus no complication	12%
Anemia	11.93%

The top 10 most prevalent diseases among the 8 million samples; it shows the percentage of the samples having that disease.

**Table 3:** Some of the most imbalanced diseases categories

Disease Category	Percent of Active class
Male Genital Disease	0.01%
Testis Cancer	0.046%
Encephalitis	0.059%
Aneurysm	0.74%
Breast Cancer	1.66%
Peripheral Atherosclerosis	3.16%
Diabetes Mellitus w/complication	4.7%

One limitation of the 2005 HCUP data set is the arbitrary order in which the ICD-9 codes and disease categories were listed. The codes were not listed in the chronological order according to the date they were diagnosed. Also, the data set does not provide anonymous patient identifier, which could be used to check if multiple records belong to the same patient or to determine the elapsed time between diagnoses.

### Data Pre-processing

The data set was provided in a large ASCII file containing the 7,995,048 records. The first step was to parse the data set, randomly select *N *records and extract a set of relevant features. Every record is a sequence of characters that are not delimited. However, the data set instructions specifies the starting column and the ending column in the ASCII file for each data element (length of data element). HCUP provides a SAS program to parse the data set, but we chose to develop our own program to perform the parsing.

### Feature Selection

For every record, we extracted the age, race, sex and 15 diagnosis categories. Every record is represented as a *d *= 262 dimensional feature vector. Features 1-259 are binary, one for each disease category. The remaining three features are age, race and sex. We denote the samples that contain a given disease category as "active" and the remaining ones as "inactive". The active and inactive data samples are defined only from the point of view of the disease being classified. A snippet of the data set is presented in table 4. For example, in table 4, sample 1 is active for disease category 50, while sample *N *is inactive.

**Table 4:** Sample Dataset, the bolded column (Cat. 50) represents the category to predict

	**Cat**. 1	**Cat**. 2	**Cat**. 3	....	**Cat**. 50	....	Cat. 257	Cat. 258	Cat. 259	Age	Race	Sex
**Patient 1**	0	0	0	....	**1**	....	0	1	1	69	3	0
. .	. .	. .	. .	. .	. .	. .	. .	. .	. .	. .	. .	. .
**Patient *N***	1	0	0	....	**0**	....	1	0	0	55	1	1

While, in general, using only disease categories may not lead to a valid disease prediction, the approach presented in this paper needs to be seen in the larger context of our TigerPlace eldercare research [11]. By integrating large public data sets (such as the one used in this paper) with monitoring sensors and electronic health records (EHR) data, we can achieve the required prediction precision for an efficient delivery of tailored medical information.

### Learning from Imbalanced Data

A data set is class-imbalanced if one class contains significantly more samples than the other. For many disease categories, the unbalance rate ranges between 0.01-29.1% (that is, the percent of the data samples that belong to the active class). For example, (see table 3) only 3.16% of the patients have Peripheral Atherosclerosis. In such cases, it is challenging to create an appropriate testing and training data sets, given that most classifiers are built with the assumption that the test data is drawn from the same distribution as the training data [12].

Presenting imbalanced data to a classifier will produce undesirable results such as a much lower performance on the testing that on the training data. Among the classifier learning techniques that deal with imbalanced data we mention oversampling, undersampling, boosting, bagging and repeated random sub-sampling [13,14]. In the next section we describe the repeated random sub-sampling method that we employ in this paper.

### Repeated Random Sub-Sampling

Repeated random sub-sampling was found to be very effective in dealing with data sets that are highly imbalanced. Because most classification algorithms make the assumption that the class distribution in the data set is uniform, it is essential to pay attention to the class distribution when addressing medical data. This method divides the data set into active and inactive instances, from which the training and testing data sets are generated. The training data is partitioned into sub-samples with each sub-sample containing an equal number of instances from each class, except for last sub-sample (in some cases). The classification model is fitted repeatedly on every sub-sample and the final result is a majority voting over all the sub-samples.

In this paper we used the following repeated random sub-sampling approach. For every target disease we randomly choose *N *samples from the original HCUP data set. The *N *samples are divided into two separate data sets, *N_1 _*active data samples and *N_0 _*inactive data samples, where *N_1+ _N_0 = _N*. The testing data will contain 30% active samples *N_1 _*(*TsN_1_*) while the remaining 70% will be sampled from the *N_0 _*(*TsN_0_*) inactive samples. The 30/70 ratio was chosen by trial-and-error. The training data set will contain the remaining active samples (*TrN_1_*) and inactive samples (*TrN_0_*).

**Figure 3:**
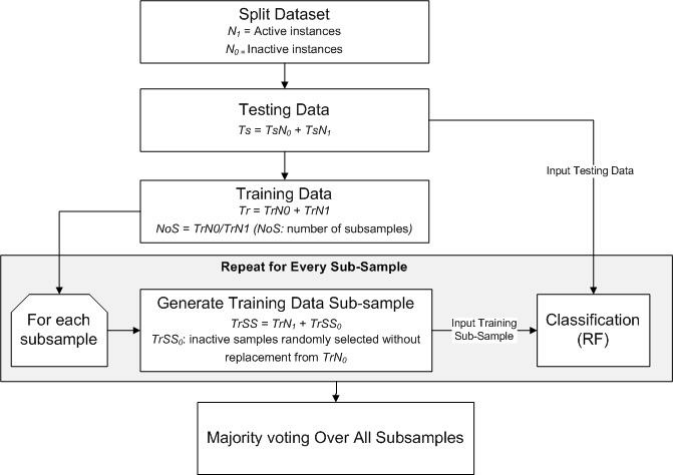
**Flow diagram of random forest and sub-sampling approach**.

Since the training data is highly imbalanced (*TrN_1 _*>>*TrN_0_*), the *TrN_0 _*samples are partitioned into *NoS *training sub-samples, where *NoS *is the ratio between *TrN_0 _*and *TrN_1_*. Every training sub-sample has equal number of instances of each class. The training active samples (*TrN_1_*) are fixed among all the training data sub-samples, while the inactive samples will be sampled without replacement from *TrN_0_*. There will be *NoS *sub-samples to train the model on. Eventually, every inactive sample in the training data is selected once, while every active sample is selected *NoS *times. After training the model on all the sub-samples, we employ a "majority voting" approach to determine the final class memberships (see Algorithm 1 in Additional File 1: Appendix). A diagram describing the process of RF and the sub-sampling procedure is presented in Figure 3.

### Random Forest

RF is an ensemble learner, a method that generates many classifiers and aggregates their results. RF will create multiple classification and regression (CART) trees, each trained on a bootstrap sample of the original training data and searches across a randomly selected subset of input variables to determine the split. CARTs are binary decision trees that are constructed by splitting the data in a node into child nodes repeatedly, starting with the root node that contains the whole learning sample [15]. Each tree in RF will cast a vote for some input *x*, then the output of the classifier is determined by majority voting of the trees (algorithm 2 in Additional File 1: Appendix). RF can handle high dimensional data and use a large number of trees in the ensemble. Some important features of RF are [16]:

1. It has an effective method for estimating missing data.

2. It has a method, weighted random forest (WRF), for balancing error in imbalanced data.

3. It estimates the importance of variables used in the classification.

Chen et al. [17], compared WRF and balanced random forest (BRF) on six different and highly imbalanced data sets. In WRF, they tuned the weights for every data set, while in BRF, they changed the votes cutoff for the final prediction. They concluded that BRF is computationally more efficient than WRF for imbalanced data. They also found that WRF is more vulnerable to noise compared to BRF. In this paper, we used RF without tuning the class weights or the cutoff parameter.

### Splitting Criterion

Like CART, RF uses the Gini measure of impurity to select the split with the lowest impurity at every node [18]. Gini impurity is a measure of the class label distribution in the node. The Gini impurity takes values in [0, 1], where 0 is obtained when all elements in a node are of the same class. Formally, the Gini impurity measure for the variable *X *= {*x*_1_, *x*_2_, ..., *x*_j_} at node *t*, where *j *is the number of children at node *t*, *N *is the number of samples, *n_ci _*is the number of samples with value *x_i _*belonging to class *c*, *a_i _*is the number of samples with value *x_i _*at node *t*, then the Gini impurity is given by [15](1)

The Gini index of a split is the weighted average of the Gini measure over the different values of variable *X*, which is given by(2)

The decision of the splitting criterion will be based on the lowest Gini impurity value computed among the *m *variables. In RF, each tree employs a different set of *m *variables to construct the splitting rules.

### Variable Importance

One of the most important features of RF is the output of the variable importance. Variable importance measures the degree of association between a given variable and the classification result. RF has four measures for the variable importance: raw importance score for class 0, raw importance score for class 1, decrease in accuracy and the Gini index. To estimate variable importance for some variable *j*, the out-of-bag (OOB) samples are passed down the tree and the prediction accuracy is recorded. Then the values for variable *j *are permuted in the OOB samples and the accuracy is measured again. These calculations are carried out tree by tree as the RF is constructed. The average decrease in accuracy of these permutations is then averaged over all the trees and is used to measure the importance of the variable *j*. If the prediction accuracy decreases substantially, then that suggests that the variable *j *has strong association with the response [19]. After measuring the importance of all the variables, RF will return a ranked list of the variable importance.

Formally, let *β_t _*be the OOB samples for tree *t*, *t *∈ {1,..., *ntree*}, *y'^t^_i _*is the predicted class for instance *i *before the permutation in tree *t *and *y'^t^_i,α _*is the predicted class for instance *i *after the permutation. The variable importance *VI *for variable *j *in tree *t *is given by(3)

The raw importance value for variable *j *is then averaged over all trees in the RF.(4)

The variable importance used in this paper is the Mean Decrease Gini (MDG), which is based on the Gini splitting criterion discussed earlier. The MDG measure the decrease *ΔI *(equation 1) that results from the splitting. For two class problem, the change in *I *(equation 6) at node *t *is defined as the class impurity (equation 5) minus the weight average of Gini measure (equation 2) [20,21].(5)(6)

The decrease in Gini impurity is recorded for all the nodes *t *in all the trees (*ntree*) in RF for all the variables and Gini Importance (*GI*) is then computed as [20].(7)

### Classification with Repeated Random Sub-Sampling

Training the classifier on a data set that is small and highly imbalanced will result in unpredictable results as discussed in earlier sections. To overcome this issue, we used repeated random sub-sampling. Initially, we construct the testing data and the *NoS *training data sub-samples. For each disease, we train *NoS *classifiers and test all of them on the same data set. The final labels of the testing data are computed using a majority voting scheme.

### Model Evaluation

To evaluate the performance of the RF we compared it to SVM on imbalanced data sets for eight different chronic diseases categories. Two sets of experiments were carried out:

Set I: We compared RF, boosting, bagging and SVM performance with repeated random sub-sampling. Both classifiers were fitted to the same training and testing data and the process was repeated 100 times. The ROC curve and the average AUC for each classifier were calculated and compared. To statistically compare the two ROC curves we employed an Analysis of Variance (ANOVA) approach [22] where the standard deviation of each AUC was computed as:(8)

where *C_p_*, *C_n_*, θ are the number positive instances, negative instances and the AUC, respectively and(9)

Set II: In this experiment we compare RF, bagging, boosting and SVM performance without the sampling approach. Without sampling the data set is highly imbalanced, while sampling should improve the accuracy since the training data sub-samples fitted to the model are balanced. The process was again repeated 100 times and the ROC curve and the average AUC were calculated and compared.

## Results

**Figure 4:**
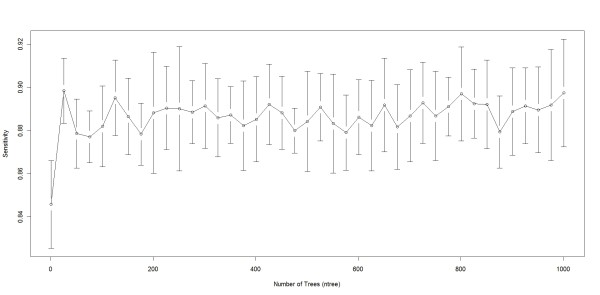
**RF behaviour when the number of trees (*ntree*) varies**. This plot shows how sensitivity in RF varies as the number of trees (*ntree*) varies, we varied *ntree *from 1-1001 in intervals of 25 and measured the sensitivity at every interval. Sensitivity ranged from 0.8457 when *ntree *= 1 and 0.8984 when *ntree *= 726. In our experiments we used *ntree *= 500 since the *ntree *did not have a large affect on accuracy for *ntree *>1.

We performed the classification using R, which is open source statistical software. We used R Random Forest (randomForest), bagging (ipred), boosting (caTools) and SVM (e1071) packages. There are two parameters to choose when running a RF algorithm: the number of trees (*ntree*) and the number of randomly selected variables (*mtry*). The number of trees did not significantly influence the classification results. This can be seen in Figure 4, where we ran RF for different *ntree *values to predict breast cancer. As we see from Figure 4, the sensitivity of the classification did not significantly change once *ntree*>20. The number of variables randomly sampled as candidates at each split (*mtry*) was chosen as the square root of the number of features (262 in our case), hence *mtry *was set to 16. Palmer et al. [23] and Liaw et al. [24] also reported that RF is usually insensitive to the training parameters.

For SVM we used a linear kernel, termination criterion (*tolerance*) was set to 0.001, *epsilon *for the insensitive-loss function was 0.1 and the regularization term (*cost*) was set to 1. Also, we left bagging and boosting with the default parameters.

We randomly selected *N *= 10,000 data points from the original HCUP data set. We predicted the disease risks on 8 out of the 259 disease categories. Those categories are: breast cancer, type 1 diabetes, type 2 diabetes, hypertension, coronary atherosclerosis, peripheral atherosclerosis, other circulatory diseases and osteoporosis.

### Result set I: Comparison of RF, bagging, boosting and SVM

**Figure 5:**
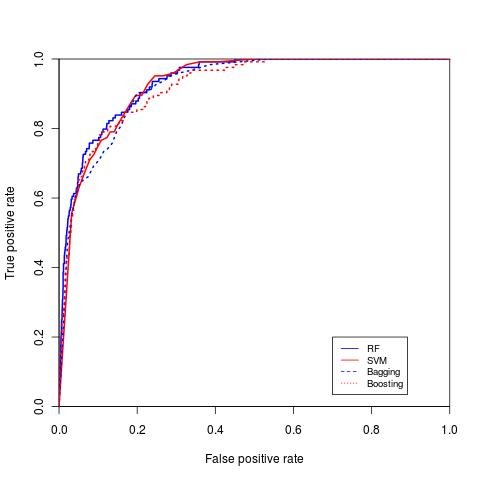
**ROC curve for diabetes mellitus**. ROC curve for diabetes mellitus comparing SVM, RF, boosting and bagging.

**Figure 6:**
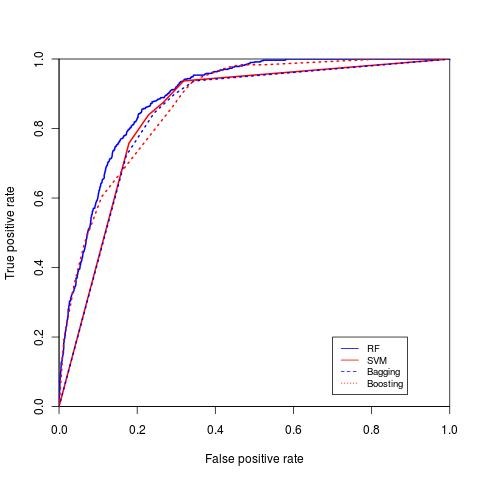
**ROC curve for hypertension**. ROC curve for hypertension comparing both SVM, RF, boosting and bagging.

**Figure 7:**
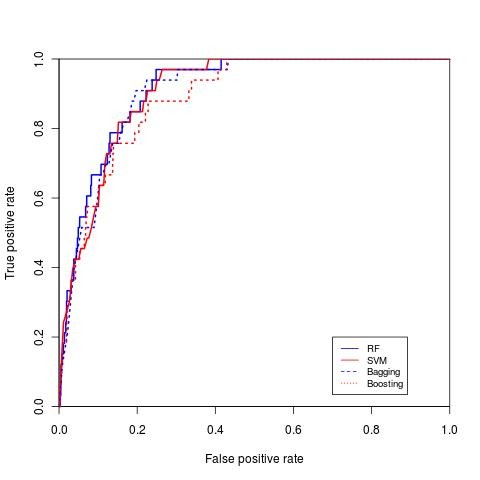
**ROC curve for breast cancer**. ROC curve for breast cancer comparing both SVM, RF, boosting and bagging.

RF, SVM, bagging and boosting classification were performed 100 times and the average area under the curve (AUC) was measured (ROC curves for the four classifiers for diabetes with complication, hypertension and breast cancer are shown in Figure 5, Figure 6 and Figure 7, respectively). The repeated random sub-sampling approach has improved the detection rate considerably. On seven out of eight disease categories RF outperformed the other classifiers in terms of AUC (table 5). In addition to ROC comparison, we used ANOVA [22] as mentioned earlier to statistically compare the ROC of boosting and RF, since both of these classifiers scored the highest in terms of AUC. ANOVA results comparing RF ROC and boosting ROC are summarized in table 6. The lower the *p *value is the more significant the difference between the ROCs is. The results of ANOVA test tells us that although RF outperformed boosting in terms of AUC, that performance was only significant in three diseases only (high prevalence diseases). The possible reason for performance difference insignificance for the other 5 diseases (mostly low prevalence diseases) might be the low number of active samples available in our sampled dataset. For example, for breast cancer we would have about 166 cases available.

**Table 5:** RF, SVM, bagging and boosting performance in terms of AUC on eight disease categories

Disease	RF	SVM	Bagging	Boosting
Breast cancer	**0.9123**	0.9063	0.905	0.8886
Diabetes no complication	**0.8791**	0.8417	0.8568	0.8607
Diabetes with/complication	**0.94317**	0.9239	0.9294	0.9327
Hypertension	**0.9003**	0.8592	0.8719	0.8842
Coronary Atherosclerosis	**0.9199**	0.8973	0.887	0.9026
Peripheral Atherosclerosis	**0.9095**	0.8972	0.8967	0.9003
Other Circulatory Diseases	**0.7899**	0.7591	0.7669	0.7683
Osteoporosis	**0.87**	0.867	0.8659	0.8635

**Table 6:** Statistical comparison of RF and boosting ROC curves, the lower the value the more significant the difference is

Disease	*p *value
Breast cancer	0.8057
Diabetes no complication	0.3293
Diabetes with/complication	0.6266
Hypertension	0.2
Coronary Atherosclerosis	0.2764
Peripheral Atherosclerosis	0.8203
Other Circulatory Diseases	0.566
Osteoporosis	0.908

We compared our disease prediction results to the ones reported by other authors. For instance, Yu et al. [1], describes a method using SVM for detecting persons with diabetes and pre-diabetes. They used data set from the National Health and Nutrition Examination Survey (NHANES). NHANES collects demographic, health history, behavioural information and it may also include detailed physical, physiological, and laboratory examinations for each patient. The AUC for their classification scheme I and II was 83.47% and 73.81% respectively. We also predicted diabetes with complications and without complications and the AUC values were 94.31% and 87.91% respectively (Diabetes without complication ROC curve in Figure 5).

Davis et al. [8] used clustering and collaborative filtering to predict disease risks of patients based on their medical history. Their algorithm generates a ranked list of diseases in the subsequent visits of that patient. They used an HCUP data set, similar to the data set we used. Their system predicts more than 41% of all the future diseases in the top 20 ranks. One reason for their low system performance might be that they tried to predict the exact ICD-9 code for each patient, while we predict the disease category.

Zhang et al. [10] performed classification on breast cancer metastasis. In their study, they used two published gene expression profiles. They compared multiple methods (logistic regression, SVM, AdaBoost, LogitBoost and RF). In the first data set, the AUC for SVM and RF was 88.6% and 89.9% respectively and for the second data set 87.4% and 93.2%. The results we obtained for breast cancer prediction for RF were 91.23% (ROC curve in Figure 7).

Mantzaris et al [9] predicted osteoporosis using multi-layer perceptron (MLP) and probabilistic neural network (PNN). Age, sex, height and weight were the input variables to the classifier. They reported a prognosis rate on the testing data of 84.9%. One the same disease, we reported an AUC for RF of 87%.

One of the important features of the RF approach is the computation of the importance of each variable (feature). We used Mean Decrease Gini (Equations 5, 6, 7) measure to achieve the variable importance (table 7). Variables with high importance have strong association with the prediction results. For example, Mantzaris et al. [9] mentioned that osteoporosis (row 8) is more prevalent in people older than 50 and occurs in women more than men and that agrees with the first and forth important variables (age and sex) reported by RF (Table 7). Another example is diabetes with complication (row 3) that often presents with fluid-electrolyte imbalance and it's incidence is inversely correlated with a normal pregnancy.

**Table 7:** Top four most importance variable for the eight disease categories

Disease	Variable 1	Variable 2	Variable 3
1. Breast cancer	Age	Sex	Secondary malignant Secondary malignant sddsmalignant malignant
2. Diabetes no complication	Age	Hypertension	Hyperlipidemia
3. Diabetes with/complication	Age	Normal pregnancy	Fluid-electrolyte imbalance
4. Hypertension	Age	Hyperlipidemia	Diabetes without compl.
5. Coronary atherosclerosis	Age	Hypertension	Hyperlipidemia
6. Peripheral atherosclerosis	Age	Coronary Atherosclerosis	Hypertension
7. Other circulatory diseases	Age	Dysthymia	Anemia
8. Osteoporosis	Age	Race	Hypertension

### Result set II: Sampling vs. non-sampling

**Figure 8:**
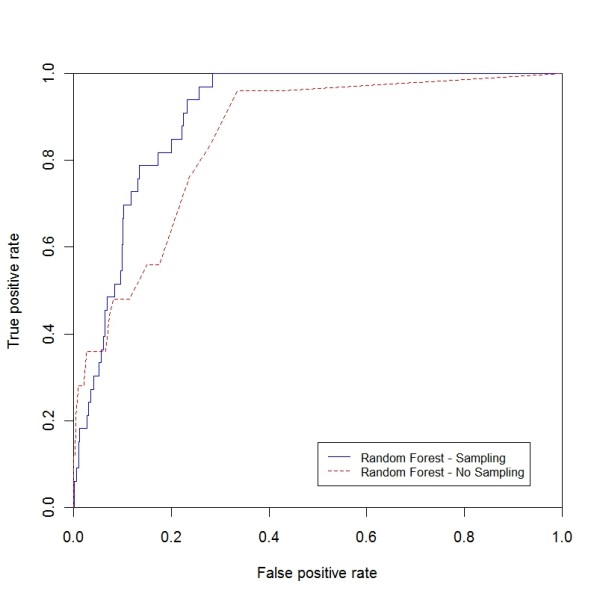
**ROC curve for breast cancer (sampling vs. non-sampling)**. ROC curve for breast cancer comparing RF with the sampling and non-sampling approach.

**Figure 9:**
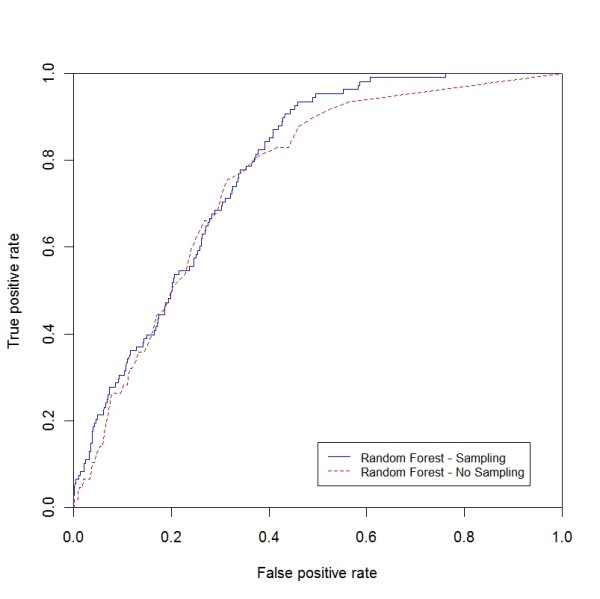
**ROC curve for other circulatory diseases (sampling vs. non-sampling)**. ROC curve for other circulatory diseases comparing RF with the sampling and non-sampling approach.

**Figure 10:**
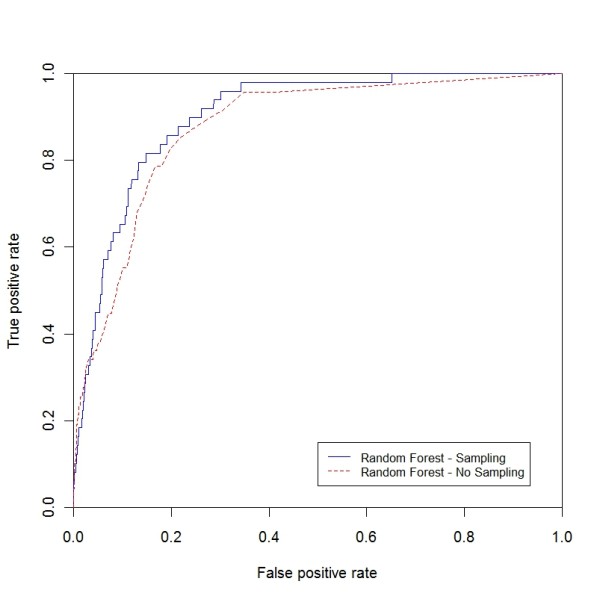
**ROC curve for peripheral atherosclerosis (sampling vs. non-sampling)**. ROC curve for peripheral atherosclerosis comparing RF with the sampling and non-sampling approach.

In this section we show that classification with sampling outperforms standalone classifiers on the HCUP data set (table 8). RF, bagging, boosting and SVM with sampling have higher ROC curves and reaches a detection rate of 100% faster than the standalone classifiers. For demonstration purposes, we included the comparisons for RF with and without sampling for three disease categories, breast cancer, other circulatory diseases and peripheral atherosclerosis (ROC curve in Figure 8, Figure 9, and Figure 10). Table 8 describes the results for the non-sampling classification for the four mentioned classifiers.

**Table 8:** RF, SVM, bagging and boostingperformance without sub-sampling in terms of AUC on eight disease categories

Disease	RF	SVMSVM	Bagging	Boosting
Breast cancer	**0.8793**	0.5	0.5085	0.836
Diabetes no complication	**0.8567**	0.5	0.4749	0.8175
Diabetes with/complication	**0.9084**	0.648	0.4985	0.8278
Hypertension	**0.8893**	0.6908	0.4886	0.8515
Coronary Atherosclerosis	**0.9193**	0.6601	0.4945	0.8608
Peripheral Atherosclerosis	**0.8872**	0.5	0.4925	0.8279
Other Circulatory Diseases	**0.7389**	0.5	0.4829	0.6851
Osteoporosis	0.7968	0.5	0.4931	**0.8561**

## Discussion

Disease prediction is becoming an increasingly important research area due to the large medical datasets that are slowly becoming available. While full featured clinical records are hard to access due to privacy issues, deidentified large public dataset are still a valuable resource for at least two reasons. First, they may provide population level clinical knowledge. Second, they allow the data mining researcher to develop methodologies for clinical decision support systems that can then be employed for electronic medical records. In this study, we presented a disease prediction methodology that employs random forests (RF) and a nation-wide deidentified public dataset (HCUP). We show that, since no national medical warehouse is available to date, using nation-wide datasets provide a powerful prediction tool. In addition, we believe that the presented methodology can be employed with electronic medical records, if available.

To test our approach we selected eight chronic diseases with high prevalence in elderly. We performed two sets of experiments (set I and set II). In set I, we compared RF to other classifiers with sampling, while in set II we compared RF to other classifiers without sub-sampling. Our results show that we can predict diseases with an acceptable accuracy using the HCUP data. In addition, the use of repeated random sub-sampling is useful when dealing with highly imbalanced data. We also found that incorporating demographic information increased the area under the curve by 0.33-10.1%.

Some of the limitations of our approach come from limitations of the HCUP data set such as the arbitrary order of the ICD-9 codes and lack of patient identification. For example, since the ICD-9 codes are not listed in chronological order according to the date they were diagnosed, we inherently use future diseases in our prediction. This explains, in part, the high accuracy of our prediction. In addition, the HCUP data set does not provide anonymous patient identifier, which can be used to check if multiple records belong to the same patient and to estimate the time interval between two diagnoses. Hence we might use the data for the same patient multiple times.

Additionally, the data set does not include the family history; rather it includes the individual diagnosis history which is represented by the diseases categories.

## Conclusions

In this study we used the NIS dataset (HCUP) created by AHRQ. Few researchers have utilized the NIS dataset for disease predictions. The only work we found on disease prediction using NIS data was presented by Davis et al. [5], in which clustering and collaborative filtering was used to predict individual disease risks based on medical history. In this work we provided extensive proof that RF can be successfully used for disease prediction in conjunction with the HCUP dataset.

The accuracy achieved in disease prediction is comparable or better than the previously published results. The average RF AUC obtained across all disease was about 89.05% which may be acceptable in many applications. Additionally, unlike many other published results were they focus on predicting one specific disease, our method can be used to predict the risk for any disease. Finally, we consider the results obtained with the proposed method adequate for our intended use, which is tailored health communication.

## Competing interests

The authors declare that they have no competing interests.

## Authors' contributions

MK is the main author of this paper. He designed and wrote the code, ran the experiments, collected the results and analyzed them, and drafted the manuscript. Professor SC developed and contributed to the design of the experiments, specially the repeated random sub-sampling approach and reviewed the manuscript. MP, MK's advisor, provided the data, helped in designing the experiments, provided continuous feedback on the paper, mainly the result section and reviewed the manuscript.

All authors have read and approved the final manuscript.

## Pre-publication history

The pre-publication history for this paper can be accessed here:

http://www.biomedcentral.com/1472-6947/11/51/prepub

## Supplementary Material

Additional file 1 **Appendix. This file contains two algorithms**. Algorithm 1, which describes the repeated random sub-sampling and algorithm 2 which briefly explains the general random forest for classification. Click here for file
