# Supplementary material for: Predicting disease risks from highly imbalanced data using random forest
Source: BMC Med Inform Decis Mak. 2011 Jul 29;11:51. doi: 10.1186/1472-6947-11-51 (PMC3163175; doi:10.1186/1472-6947-11-51)
Supplement: Additional file 1 — Appendix. This file contains two algorithms. Algorithm 1, which describes the repeated random sub-sampling and algorithm 2 which briefly explains the general random forest for classification. [file 1472-6947-11-51-S1.DOC]

| **Algorithm 1** Repeated Random Sub-Sampling |
| --- |
| 1. *TsN* = total number of samples in the testing data 2. *N_0_* = number of inactive samples 3. *N_1_* = number of active samples 4. *N* = total number of samples in the data set, where *N*=*N_0_*+*N_1_* 5. Generate testing data    1. Randomly select *TsN1* samples from *N1* , where *TsN1*=0.3**N1*    2. Randomly select *TsN0* samples from *N0*, where *TsN0*=*TsN* - *TsN1* 6. *Ts* = *TsN0* samples + *TsN1* samples (*Ts* = testing data) 7. Generate training data    1. Contains *TrN1* samples, *TrN1* = remaining *N1* samples after generating testing data    2. Contains *TrN0* samples, *TrN0* = remaining *N0* samples after generating testing data 8. *NoS = TrN0/TrN1* 9. **for** *s* = 1 to *NoS* **do**    1. Generate the *s* training data sub-sample       1. *TrSS0* = Randomly select *TrN1* (number of training active samples) samples from *TrN0* inactive samples without replacement (Guarantees full balance of the training data sub-samples, except for the last sub-samples, in some cases)    2. *TrSS = TrSS0 + TrN1*    3. ys(x) = classifer(*TrSS*, *Ts*) (Predicted class labels for *Ts* using sub-sample *TrSS*) 10. **end for** 11. y(x) = majority voting {y_s_(x)}*^NoS^*_1_ (Final predicted class is majority voting over all sub-samples) |

| **Algorithm 2** Random Forest for Classification |
| --- |
| 1. *ntree* = number of trees to be generated 2. *N* = number of samples in the data set 3. **for** *t* = 1 to *ntree* **do**    1. Generate bootstrap sample *Z* of size *N* from the original data - with replacement    2. for each bootstrap *Z* grow a classi_cation tree    3. for *i* = 1 to *NumberOfNodes* do       1. randomly sample *mtry* variables from *M* variables       2. choose best split among the sampled variables (bagging is special case of RF and obtained when*mtry* = *M*)    4. end for    5. yt(x) = class prediction of the *t*th tree 4. **end for** 5. Yrf (x) = majority voting {y_t_(x)}*^ntree^*_1_ (Final predicted class is majority voting over all trees in RF) |
